# Useful Hints to Hospitals

**Published:** 1844-11

**Authors:** 


*Useful Hints to Hospitals.*—In the hospital at Frankfort, I noticed a peculiarity in the make of the mattresses which deserves to be noticed. They consist of three portions or compartments, which are perfectly joined into one when the bed is made; but which are readily separated from each other, so that any part may be removed and cleaned at a time without the others. As a matter of course, it is the middle one that generally requires to be so treated. By this simple arrangement, the mattresses may be, at a very small expense, always kept in cleanliness and order.—*Med. Chir. Review.*

